# Management of patients with retroperitoneal tumors and a review of the literature

**DOI:** 10.1186/s12957-015-0548-z

**Published:** 2015-04-09

**Authors:** Kazım Gemici, İbrahim Buldu, Türker Acar, Hüsnü Alptekin, Mehmet Kaynar, Erdem Tekinarslan, Tuna Karatağ, Duran Efe, Haldun Çolak, Tevfik Küçükkartallar, Mustafa Okan İstanbulluoğlu

**Affiliations:** Faculty of Medicine, Department of General Surgery, Mevlana University, Konya, Turkey; Faculty of Medicine, Department of Urology, Mevlana University, Konya, Turkey; Faculty of Medicine, Department of Radiology, Mevlana University, Konya, Turkey; Faculty of Medicine, Department of Gynaecology, Mevlana University, Konya, Turkey; Konya Education and Research Hospital, Konya, Turkey; Faculty of Medicine, Department of General Surgery, Necmettin Erbakan University, Konya, Turkey

**Keywords:** Liposarcoma, Retroperitoneal, Resection, Mesenchymal tumors

## Abstract

**Background:**

Retroperitoneal tumors (RTs) develop insidiously and are generally seen as large masses, and 50% of RTs are larger than 20 cm at the time of diagnosis. In this article, we share our experience of 5 years of surgical management of RTs.

**Methods:**

We evaluated 28 RT cases operated on in three education hospitals in Turkey from January 2008 onwards, with regard to patients’ demographic characteristics, complaints, weight loss figures, the location and size of the tumor, blood transfusion, intra-operational time, metastases (in malignant cases), additional organ resection, histological grade, local recurrences, average life expectancy, and post-operative treatment methods.

**Results:**

The mean age of the patients was 49 years (range, 18 to 78 years). Twenty (71.43%) were female, and 8 (28.57%) were male. The primary complaint was abdominal pain in 18 patients (64.28%). CT scans were performed in 17 (61%) patients, 10 (35.4%) underwent abdominal MR imaging, and 1 (3.6%) underwent both abdominal CT and abdominal MR imaging. A mass was palpated in the pelvis (suprapubic region) in seven (25%) of the patients during physical examination. The largest tumors were detected in the left lumbar area. The mean tumor size was 12.78 cm (range, 2 to 30 cm). The mean intra-operational time was 192 min (range, 70 to 380 min). The mean hospitalization period was 11 days (range, 8 to 23 days). Seven (25%) patients were reported to have benign tumors, while 21 (75%) were reported to have malignant tumors. The most frequently seen malignant pathology was liposarcoma (eight cases; 38.09%) followed by leiomyosarcoma (five cases; 23.8%) and malignant fibrous histiocytoma (four cases; 19.04%). The earliest local recurrence was detected in the 12th month and the latest in the 28th month. A total of 11 (52.3%) of the total of 21 malignant cases experienced local recurrence within 3 years. The 3-year average life expectancy was 85.7% in the 18 malignant cases.

**Conclusions:**

Due to the low response rate of all but two types of RT to chemotherapy, the best remaining treatment option is surgery with wide resection margins, whereby all macroscopic traces of tumor are removed.

## Background

Retroperitoneal tumors (RTs) commonly present with abdominal distention and palpable mass. In many cases, they are detected as a result of imaging techniques performed to investigate unrelated issues. Although RTs can be located in the gastrointestinal and urinary tracts, patients rarely present with symptoms in these systems [1,2]. The retroperitoneal space is the second most frequent location, followed by the lower extremities, where malignant mesenchymal tumors arise. Each year, approximately 250 to 300 new cases of retroperitoneal sarcoma are diagnosed in the United Kingdom [3]. Despite the rare nature of RTs, two thirds of these diagnoses represent malignant tumors. Approximately, one third of RT cases are sarcomas. The most frequent sarcomas are liposarcoma, malignant fibrous histiocytoma, and leiomyosarcoma, respectively. Other malignant RT types are lymphoma, epithelial tumors, malignant paraganglioma (which is considered to be benign when no metastasis occurs), and metastatic tumors. Fibromatosis, renal angiomyolipoma, benign paraganglioma, neurofibroma, lipoma, angiofibroma, and schwannoma can be listed among the benign tumors. The majority of sarcomas in the RT region cannot be completely removed surgically because of their close proximity to vital organs (in contrast to tumors located in the extremities). Despite this, surgery remains the most successful treatment method for RTs, significantly affecting post-operative survival [4]. RTs develop insidiously and are generally seen as large masses; 50% of RT is larger than 20 cm at the time of diagnosis. RTs develop without suppressing the inner organs or causing significant lumen blockage and are frequently confused with lymphomas. Full physical examination, evaluation of all peripheral lymph nodes, and testis examination for male patients are important when approaching patients with RT. RT liposarcomas are most frequently seen between 50 and 70 years of age. With a 5-year survival rate of between 40% and 50% [5], the prognosis is worse than that of other soft tissue sarcomas regarding local recurrence. The goal in surgical management of RTs is to achieve the optimal negative surgical border. In the presence of a positive surgical border, the 5-year survival rate decreases to 28%. Delayed diagnosis, high histological grade, inoperability due to invasion into vital organs, and a positive surgical border can be listed among the most significant factors affecting survival. The average life expectancy for patients with high-grade RTs is 20 months, while for low-grade RTs, it is 80 months; moreover, RTs larger than 10 cm generally had distant metastasis at the time of diagnosis [6].

## Methods

Twenty-eight patients diagnosed with RT were operated on at three distinct education hospitals in Turkey between 2008 and 2013. Written informed consent was obtained from each individual before surgical procedure. Patients were followed-up for 3 years. The patients’ clinical records, radiology reports, and pathology reports were evaluated, and their demographic characteristics, complaints, amount of weight loss, the location and size of tumors as right upper quadrant (RUQ), right lower quadrant (RLQ), suprapubic (SP), left lower quadrant (LLQ), and left upper quadrant (LUQ) were determined. In addition, incidences of blood transfusion, operation time, metastases if detected, additional organ resections, histological grades, local recurrences, hospitalization periods, average life expectancy, and post-operative treatments of the patients were analyzed. Wide resection (WR) with safe margins was performed in patients with a single tumor while WR covering both the main tumor and any nodules was performed in patients with nodular spread in addition to the main tumor. Wound infection, wound separation, hemorrhage, pneumonia, re-operation, sepsis, intra-abdominal abscess, ileus, deep vein thrombosis, and enterocutaneous fistula were observed to be the most important post-operative complications. RT sizes were determined by taking the maximum diameters into consideration. The patients were called in for follow-up every 3 months in the first year and every 6 months in the second and third years, in the absence of complaints. Abdominal CT or MR imaging was performed during the 6-month follow-up analyses.

## Results

**Figure 1:**
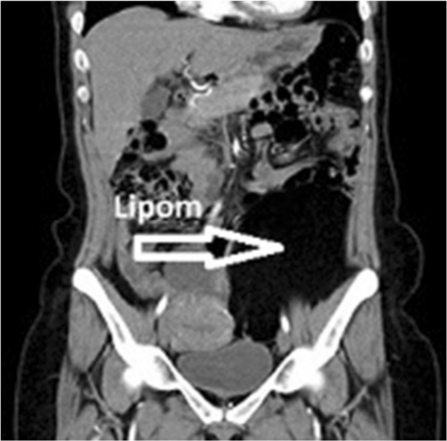
**CT image of lipoma in the lower left quadrant.**

**Figure 2:**
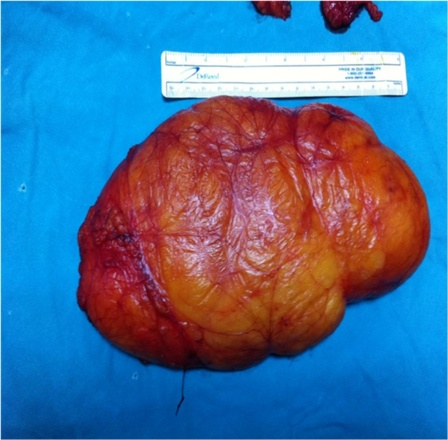
**Appearance of lipoma (15 cm).**

**Figure 3:**
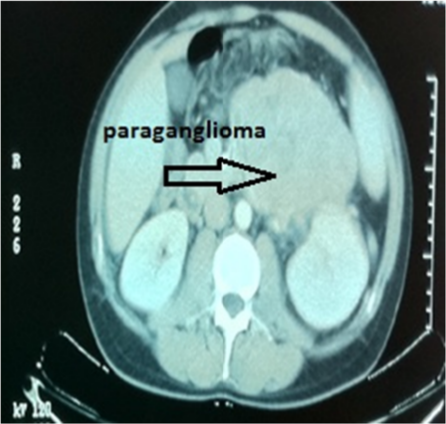
**Pre-operative CT image of malign paraganglioma.**

**Figure 4:**
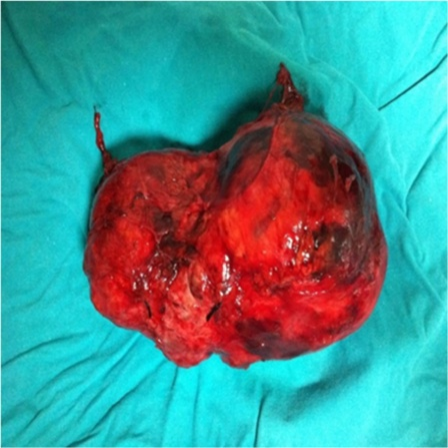
**Post-operative sight of a malign paraganglioma (17 cm).**

**Figure 5:**
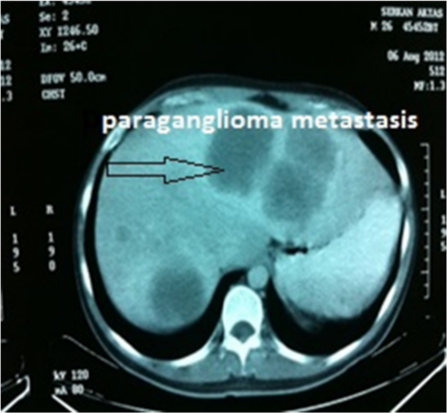
**CT image of paraganglioma at the sixth months.**

Table 1 presents patients’ demographic characteristics, tumor grades, tumor sizes, additional organ resections, and features of wezight loss. The mean hospitalization period was 11 days (ranging from 8 to 23 days). While 20 (71.4%) of the patients were female, 8 (28.6%) were male. The mean age-age ranges for female and male patients were 55 (28 to 78) and 38 (18 to 54) years, respectively. Average weight loss in the last 6 months was 4.3 (±1.2) kg. The primary complaints of the patients were abdominal pain in 18 patients (64.28%), abdominal induration in 9 (32.14%), and loss of strength in the right foot in 1 (3.58%) patient. The beginning of the complaints could be traced back an average of 8 months. Twenty-one (75%) of the RTs were malignant, while 7 (25%) were benign (Figures 1 and 2), and the most frequent pathologies were liposarcoma in 8 (38.1%) patients, leiomyosarcoma in 5 (23.8%), malignant fibrous histiocytoma in 5 (23.8%), and malignant paraganglioma in 1 (4.76%) patient who had generalized intra-abdominal metastasis in the sixth month (Table 2). The same patient was lost in the second year (Figures 3, 4 and 5).

**Table 1:** **Characteristics of the patients and tumors**

**Characteristics**	**Value**
Age (*n* = 28)	
Male	38 (18 to 54)
Female	55 (28 to 78)
Gender (*n*, %)	
Male	8 (28.6%)
Female	20 (71.4%)
Grade (*n* = 21)	
Grade 1	10 (47.61%)
Grade 2	7 (33.33%)
Grade 3	4 (19.06%)
Tumor size (*n* = 28)	
≤10 cm	13 (46.42%)
11 to 20 cm	11 (39.28%)
>20 cm	4 (14.3%)
Additional organ resection	
Yes	5 (17.85%)
No	23 (82.15%)
Weightloss^a^	4.3 (±1.2) kg

^a^In the last 6 months.

**Table 2:** **Histopathological distribution of RTs**

**Tissue type**	**Number (%)**
Malignant (*n* = 21)	
Lyposarcoma	8 (38.09%)
Leiomyosarcoma	5 (23.80%)
Malign fibrous histiocytoma	5 (23.80%)
Malign paraganglioma	1 (4.76%)
Hodgkins lymphoma	1 (4.76%)
Fibrosarcoma	1 (4.76%)
Benign (*n* = 7)	
Neurofibroma	2 (28.57%)
Lipoma	1 (14.28%)
Fibroma	1 (14.28%)
Angiofibroma	1 (14.28%)
Schwannoma	1 (14.28%)
Benign paraganglioma	1 (14.28%)

In three (10.7%) cases, wound infection was experienced; these patients recovered in an average of 6 days through local drainage and appropriate antibiotic treatment based on culture antibiogram. Two (7.1%) patients suffered from ileus, recovering within 3 to 7 days following nasogastric decompression and medical treatment. One (3.5%) obese patient had the complication of pneumonia, which was confirmed radiologically and through culture; this patient’s clinical and radiological symptoms regressed after 15 days of antibiotic treatment.

CT scans were performed in 17 (61%) patients, 10 (35.4%) had abdominal MR imaging, and 1 (3.6%) had both abdominal CT and abdominal MR imaging. A mass was palpated in the pelvis (suprapubic region) of seven (25%) patients during physical examination, with the second most frequent area of mass palpation in the left upper quadrant in six (21.4%) patients. The least frequent area of observation of a mass was the right upper quadrant, in one (3.57%) patient.

**Figure 6:**
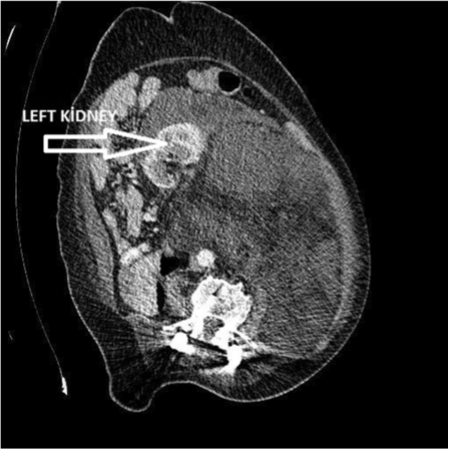
**CT image of left kidney being pushed laterally and anteriorly to the right.**

The largest tumors were detected in the left lumbar area. The smallest tumor was in the right upper quadrant. The average tumor size in malignant cases with no macroscopic metastases was 10.5 cm, and the most frequent spread was in nodular form besides the main tumor in eight (38.1%) patients in the malignant group (*n* = 21) (Table 3). Patients who had isolated tumors with no nodular spread underwent wide resection. Those with nodular spread had wide resection both to the main tumor and the nodules. Wide resection and splenectomy were performed in one patient with spleen metastasis. One (4.76%) patient, in whom spleen and stomach invasion was detected, underwent wide resection plus splenectomy and stomach wedge resection. The location of intra-abdominal organs can shift in the presence of RTs, due to the effect of the mass, with the kidneys being the most common viscera with an altered intra-abdominal location. In our series, one RT (longest length measuring 30 cm) pushed the left kidney to the right anterolateral direction (Figure 6). Since the left kidney was completely engulfed by the mass and many nodular tumor implants were detected during imaging work-up, the patient underwent multiple WR (MWR) and a left nephrectomy. An incision biopsy procedure was performed in one distinct patient with aortic invasion, and the result was reported to be grade 3 liposarcoma. Biopsy was performed in one patient with aorta and vena cava inferior invasion and the result was Hodgkin’s lymphoma (mixed type). Wide resection and left hemicolectomy were performed in one patient with left colon invasion (Table 4). The earliest local recurrence was detected in the 12th month and the latest in the 28th month. A total of 11 (52.3%) of a total of 21 malignant cases experienced local recurrence within 3 years. The 3-year average life expectancy was 85.7% in 18 patients among the malignant cases. Three (14.3%) patients died due to widespread intra-abdominal recurrence and hepatic metastasis, while one patient was lost because of lung metastasis in addition to generalized metastasis. Ten (47.61%) patients with satellite nodules and visceral involvement received post-operative radiotherapy. Five (23.8%) patients received chemotherapy (CT) in addition to radiotherapy (RT).

**Table 3:** **The localizations of the tumors and the sizes of the malignant tumors according to metastasis status**

**Region**	***n*** **(%)**	**Mean tumor size (cm)**	**Metastasis status**	**Number = 21 (%)**	**Size (cm) mean**
RUQ	1 (3.57%)	2	Nodular	5 (23.80%)	14.2
RIQ	2 (7.14%)	10	Liver + Nodular	1 (4.76%)	16
LUQ	6 (21.42%)	13.3	Right tubo-ovarian	1 (4.76%)	6
LIQ	6 (21.42%)	8.8	+ Nodular		
LLR	3 (10.71%)	23.3	Left kidney + Nodular	1 (4.76%)	30
SNT	3 (10.71%)	17.3	Spleen, stomach	1 (4.76%)	17
SP	7 (25%)	11.5	Aortic	1 (4.76%)	24
			Aortic, VCI	1 (4.76%)	10
			Colon	1 (4.76%)	8
			Spleen	1 (4.76%)	15
			None	8 (38.1%)	10.5

RUQ: Right upper quadrant, RIQ: Right inferior quadrant, LUQ: Left upper quadrant, LIQ: Left inferior quadrant, LLR: Left lumbar region, SNT: Periumblical region, SP: Suprapupic region, VCI: Vena cava inferior.

**Table 4:** **Characteristics of all surgical procedures**

**Patient**	**Tumor size (cm)**	**Operation**	**Histology**	**Grade**	**Operation time**	**Blood Tx (units)**
1	12	WTR	LS	G1	160	1
2	20	WTR	LMS	G1	200	2
3	6	MWTR + RSO	FS	G2	235	1
4	8	WTR	NF		120	-
5	17	WTR	L		280	2
6	30	MWTR	LS	G3	380	3
7	8	WTR	FR		150	-
8	12	WTR	LS	G1	210	2
9	16	WTR	LMS	G1	280	1
10	19	MWTR	LMS	G2	190	-
11	30	MWTR + LN	LS	G2	230	4
12	15	WTR + SP	PRG	G3	240	3
13	25	WTR	ANGF		320	2
14	6	MWTR	LMS	G1	140	-
15	11	MWTR	MFH	G2	180	-
16	7	WTR	SWN		110	-
17	8	WTR	NF		200	2
18	5	MWTR	MFH	G1	100	1
19	3	WTR	LS	G1	90	1
20	16	MWTR^a^	LS	G2	215	-
21	4	WTR	LMS	G1	130	-
22	17	WTR + SP + WRS	MFH	G2	360	5
23	15	WTR	LS	G1	225	1
24	24	BPY^b^	LS	G3	70	-
25	2	WTR	MFH	G1	115	3
26	8	WTR + LHC	MFH	G2	260	7 + 4 FFP
27	4	WTR	PRG		120	1
28	10	BPY^c^	HL	G3	70	-

LS: Liposarcoma, LMS: Leiomyosarcoma, MFH: Malignant fibrous histiocytoma, FS: Fibrosarcoma, PRG: Paraganglioma, L: Lipoma, NF: Neurofibroma, FR: Fibroma, ANGF: Angiofibroma, SWN: Schwannoma, HL: Hodgkin’s Lymphoma , WTR: Wide total resection, MWTR: Multiple wide total resection (applied for the patients with nodular progression), RSO: Right salpingo-oophorectomy, LN: Left Nephrectomy, SP: Splenectomy, LHC: Left hemicolectomy, WRS: Wedge resection of the stomach, BPY: Biopsy, FFP: Fresh frozen plasma. ^a^Liver metastases; ^b^aortic invasion; ^c^aortic and vena cava invasion.

**Figure 7:**
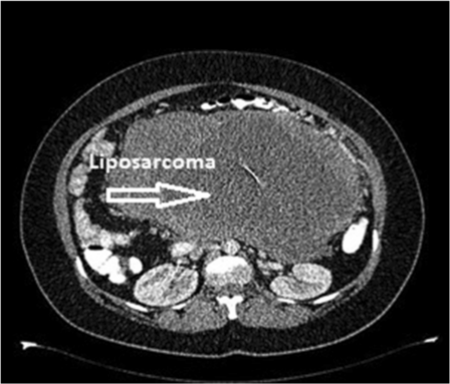
**CT image of grade 3 liposarcoma in size of 30 cm (pleomorphic type).**

The mean operation time was 192 min (ranging from 70 to 380 min). One patient received 7 U erythrocyte suspension and 4 U fresh frozen plasma (FFP) due to massive hemorrhage during the operation. The average amount of blood needed was 1.5 U in our series. A patient whose pathology was reported to be grade 3 liposarcoma, which is presented as the sixth item in Table 4, received wide resection with safe margins including satellite nodules, removing all macroscopic tumor traces during the procedure; the patient also received post-operative CT plus RT (Figure 7). When the patient presented to their third follow-up in the 12th month, abdominal ultrasonography (USG) revealed multiple widespread nodular implants, the largest of which was 16 cm, and widespread liver and lung metastases. This patient was lost in the 15th month. The patient with colon invasion was also lost in the 25th month due to widespread abdominal and hepatic metastasis.

## Discussion

RTs are challenging for surgeons due to their inaccessible location, unpredictable clinical behavior, and lack of successful treatments. A diagnosis can be reached through the evaluation of complaints, clinical findings, imaging methods, and Trucut biopsy. The most common tumors among the malignant group are liposarcomas. These tumors generally tend to be of low and intermediate grades. In it has been reported that the following are negative prognostic factors: poorly differentiated type, grade 2 to 3 tumor, stage 2 to 3 tumor, tumor size larger than 20 cm, and a positive surgical border [7]. The well-known grading systems for sarcomas currently in use at present are those of the National Cancer Institute (NCI) and French Federation of Cancer Centers (FFCLCC) [8]. Within the framework of a study evaluating 500 patients with retroperitoneal sarcoma, who were treated at the Memorial Sloan-Kettering Cancer Center, it was found that while the average life expectancy for patients who had complete resection was 103 months, this decreased to 18 months for patients who had incomplete resection or whose tumors were inoperable [9]. While studies have found that the 5-year average life expectancy for well-differentiated liposarcoma was 90%, this percentage decreases to 30% to 50% for pleomorphic type liposarcomas. Liposarcomas of low grade have a high tendency to locally recur, but their tendency to distantly metastasize is low. Pleomorphic liposarcomas, however, tend to have a high tendency to distantly metastasize, and this feature has been held responsible for the decrease in average life expectancy. In our series, the earliest death happened in the 15th month in a patient with grade 3 liposarcoma of pleomorphic histological type. Since poorly differentiated liposarcomas tend to invade intra-abdominal organs more frequently, additional organ resections are often needed in these cases [10]. In 2000, Linehan *et al*. studied 159 patients diagnosed with RTs and found that the increase in tumor size played a significant role in the increase of local recurrence and in metastasis rates [11].

The largest tumor size in our series was 30 cm. In patients with large tumors, if the existence of RTs is confirmed through imaging, pre-operative biopsy would not be recommended due to the risk of tumor spread during the biopsy procedure. The majority of patients with RTs have local recurrences, and 75% of sarcoma-related deaths are based on these recurrences. This should be taken into consideration in all RT cases, with the exception of soft tissue sarcomas. For instance, intra-abdominal sarcomas can be frequently confused with retroperitoneal sarcomas and if imaging results cause practitioners to suspect lymphoma, core needle biopsy should be performed [12]. Since RTs frequently invade vital organs, complete macroscopic clearing can only be achieved in 55% to 93% of the patients. In our series, additional organ resection was needed in five patients. Splenectomy was performed in one patient because of spleen invasion, splenectomy and stomach wedge resection were performed in another patient because of spleen and stomach invasion, while left hemicolectomy was performed in one patient because of left colon invasion. One patient received left nephrectomy since the left kidney had completely surrounded the mass (Figure 6), and in another patient, right salpingo-oophorectomy was indispensable because the mass invaded the right ovary and fallopian tube. Palliative surgery is recommended for patients diagnosed with low and intermediate grade RTs in the case of local recurrence to manage the symptoms and to increase the patients’ quality of life [13]. Studies conducted at Johns Hopkins University School of Medicine in 2009 with 1,365 cases and at Anderson Cancer Center in Houston in 2008 with 1,091 cases showed that the biology of tumors and complete surgical resection were the keys to treatment [14]. Liposarcomas are generally rooted in perinephric fat tissues and, as they grow, cause shifting of the kidneys’ original location. This was clearly seen in our series in Figure 6, and colon resection alongside the kidney is performed frequently in such cases [15]. There is no strong evidence for the utility of radiotherapy or chemotherapy for treatment of RTs [16]. Since there is no prospective randomized controlled study on the effectiveness of radiotherapy, most of the data are based on retrospective studies [17]. The dose and duration of radiotherapy are limited because of its toxic effect on the gastrointestinal tract. Pre-operative, intra-operative, and post-operative radiotherapy can be effective, but only in a small proportion of patients [18-21]. Radiotherapy is especially recommended for patients with high-grade tumors and those for whom complete resection is impossible [22,23]. In RT surgical procedures, the organs, which are frequently resected, in addition to the primary tumor, are the kidneys, colon, pancreas, and spleen [24]. In some tumors, such as the Ewing tumors, chemotherapy forms a significant part of the treatment, while for some particular histological types, special chemotherapeutic agents are sometimes used. These include agents such as doxorubicin and ifosfamide for the palliation of sarcomas, taxanes for angiosarcomas, gemcitabine and docetaxel for leiomyosarcomas, and trabectedin for mixoid-round cell liposarcomas and leiomyosarcomas [25]. Leiomyosarcoma and malignant fibrous histiocytoma have worse prognoses in comparison to liposarcoma. The extent of distant metastases is worse than for their local recurrences [26,27]. In cases which are histopathologically confirmed to be benign, for instance in schwannomas, surgery is performed if the patient is symptomatic; on the other hand, if there are no symptoms, the patient can be followed up through imaging following tissue diagnosis [28]. The genetic and molecular complexity of tumors needs to be elucidated to gain a better understanding of the biological behavior of these tumors and to offer better treatment options [29,30].

## Conclusions

In this study, we analyzed 28 patients diagnosed with retroperitoneal tumors, based on data retrieved from three distinct training and education hospitals in Turkey. The results we found were similar to those published in the literature. It is recommended that retroperitoneal tumors are immediately treated by an experienced team of surgeons, employing a multidisciplinary approach. The only current treatment option that is known to prolong survival in patients with these tumors is wide surgical resection. While the efficiency of pre-operative and post-operative radiotherapy and chemotherapy is still a controversial issue, wide surgical resection for treatment of RTs remains the gold standard procedure.

## Abbreviations

ANGF: angiofibroma
BPY: biopsy
FFP: fresh frozen plasma
FR: fibroma
FS: fibrosarcoma
HL: Hodgkin’s Lymphoma
L: lipoma
LHC: left hemicolectomy
LIQ: left inferior quadrant
LLR: left lumbar region
LMS: leiomyosarcoma
LN: left nephrectomy
LS: liposarcoma
LUQ: left upper quadrant
MFH: malignant fibrous histiocytoma
MWTR: multiple wide total resection (applied for the patients with nodular progression)
NF: neurofibroma
PRG: paraganglioma
RIQ: right inferior quadrant
RSO: right salpingo-oophorectomy
RTs: retroperitoneal tumors
RUQ: right upper quadrant
SNT: periumbilical region
SP: splenectomy
SP: suprapubic region
SWN: schwannoma
VCI: vena cava inferior
WRS: wedge resection of the stomach
WTR: wide total resection

## References

[1] Hughes MJ, Thomas JM, Fisher C, Moskovic EC (2005). Imaging features of retroperitoneal and pelvic schwannomas. Clin Radiol.

[2] Hueman MT, Herman JM, Ahuja N (2008). Management of retroperitoneal sarcomas. Surg Clin North Am.

[3] Weiss SW, Goldblum JR (2001). Enzinger and Weiss’s soft tissue tumors.

[4] Bauer HC, Trovik CS, Alvegård TA, Berlin O, Erlanson M, Gustafson P (2001). Monitoring referral and treatment in soft tissue sarcoma: study based on 1,851 patients from the Scandinavian Sarcoma Group Register. Acta Orthop Scand.

[5] Singer S, Antonescu CR, Riedel E, Brennan MF (2003). Histologic subtype and margin of resection predict pattern of recurrence and survival for retroperitoneal liposarcoma. Ann Surg.

[6] Jagues DP, Coit DG, Hajdu SI, Brennan MF (1990). Management of primary and recurrent soft-tissue sarcoma of the retroperitoneum. Ann Surg.

[7] Neuhaus SJ, Barry P, Clark MA, Hayes AJ, Fisher C, Thomas JM (2005). Surgical management of primary and recurrent retroperitoneal liposarcoma. Br J Surg.

[8] Eilber FC, Brennan MF, Eilber FR, Dry SM, Singer S, Kattan MW (2004). Validation of the postoperative nomogram for 12-year sarcoma-specific mortality. Cancer.

[9] Kattan MW, Leung DH, Brennan MF (2002). Postoperative nomogram for 12-year sarcoma-specific death. J Clin Oncol.

[10] Lahat G, Anaya DA, Wang X, Tuvin D, Lev D, Pollock RE (2008). Resectable well-differentiated versus dedifferentiated liposarcomas: two different diseases possibly requiring different treatment approaches. Ann Surg Oncol.

[11] Linehan DC, Lewis JJ, Leung D, Brennan MF (2000). Influence of biologic factors and anatomic site in completely resected liposarcoma. J Clin Oncol.

[12] Strauss DC, Qureshi YA, Hayes AJ, Thway K, Fisher C, Thomas JM (2010). The role of core needle biopsy in the diagnosis of suspected soft tissue tumours. J Surg Oncol.

[13] Shibata D, Lewis JJ, Leung DH, Brennan MF (2001). Is there a role for incomplete resection in the management of retroperitoneal liposarcomas?. J Am Coll Sur.

[14] Nathan H, Raut CP, Thornton K, Herman JM, Ahuja N, Schulick RD (2009). Predictors of survival after resection of retroperitoneal sarcoma: a population-based analysis and critical appraisal of the AJCC staging system. Ann Surg.

[15] Lee SY, Goh BK, Teo MC, Chew MH, Chow PK, Wong WK (2011). Retroperitoneal liposarcomas: the experience of a tertiary Asian center. World J Surg Oncol.

[16] Pawlik TM, Pisters PW, Mikula L, Feig BW, Hunt KK, Cormier JN (2006). Long-term results of two prospective trials of preoperative external beam radiotherapy for localized intermediate or high grade retroperitoneal soft tissue sarcoma. Ann Surg Oncol.

[17] Pisters PW, Ballo MT, Fenstermacher MJ, Feig BW, Hunt KK, Raymond KA (2003). Phase I trial of preoperative concurrent doxorubicin and radiation therapy, surgical resection, and intraoperative electron-beam radiation therapy for patients with localized retroperitoneal sarcoma. J Clin Oncol.

[18] Van De Voorde L, Delrue L, van Eijkeren M, De Meerleer G (2011). Radiotherapy and surgery an indispensable duo in the treatment of retroperitoneal sarcoma. Cancer.

[19] National Institute for Health and Clinical Excellence. Improving outcomes for people with sarcoma. The Manual. London: NICE; 2006.

[20] Lewis JJ, Leung D, Woodruff JM, Brennan MF (1998). Retroperitoneal soft-tissue sarcoma: analysis of 500 patients treated and followed at a single institution. Ann Surg.

[21] Hassan I, Park SZ, Donohue JH, Nagorney DM, Kay PA, Nasciemento AG (2004). Operative management of primary retroperitoneal sarcomas: a reappraisal of an institutional experience. Ann Surg.

[22] Zhou Z, McDade TP, Simons JP, Ng SC, Lambert LA, Whalen GF (2010). Surgery and radiotherapy for retroperitoneal and abdominal sarcoma: both necessary and sufficient. Arch Surg.

[23] Catton CN, O’Sullivan B, Kotwall C, Cummings B, Hao Y, Fornasier V (1994). Outcome and prognosis in retroperitoneal soft tissue sarcoma. Int J Radiat Oncol Biol Phys.

[24] Strauss DC, Hayes AJ, Thway K, Moskovic EC, Fisher C, Thomas JM (2010). Surgical management of primary retroperitoneal sarcoma. Br J Surg.

[25] Krikelis D, Judson I (2010). Role of chemotherapy in the management of soft tissue sarcomas. Expert Rev Anticancer Ther.

[26] Meis JM (1991). “Dedifferentiation” in bone and soft-tissue tumors: a histological indicator of tumor progression. Pathol Annu.

[27] Coindre JM, Mariani O, Chibon F, Mairal A, De Saint Aubain Somerhausen N, Favre-Guillevin E (2003). Most malignant fibrous histiocytomas developed in the retroperitoneum are dedifferentiated liposarcomas: a review of 25 cases initially diagnosed as malignant fibrous histiocytoma. Mod Pathol.

[28] Strauss DC, Hayes AJ, Thomas JM (2011). Retroperitoneal tumours: review of management. Ann R Coll Surg Engl.

[29] Gutierrez JC, Perez EA, Moffat FL, Livingstone AS, Franceschi D, Koniaris LG (2007). Should soft tissue sarcomas be treated at high-volume centers? An analysis of 4205 patients. Ann Surg.

[30] Chowdhury MM, Dagash H, Pierro A (2007). A systematic review of the impact of volume of surgery and specialization on patient outcome. Br J Surg.

